# Is It Useful and Necessary to Add a T2 Paravertebral Block to the Regional Anesthesia During Proximal Humeral Fracture Surgery in Elderly Patients? A Prospective and Randomized Controlled Trial

**DOI:** 10.3389/fsurg.2022.755298

**Published:** 2022-03-14

**Authors:** Xiaofeng Wang, Hui Zhang, Yongzhu Chen, Qingfu Zhang, Zhenwei Xie, Junling Liao, Wei Jiang, Junfeng Zhang

**Affiliations:** Department of Anesthesiology, Shanghai Jiao Tong University Affiliated Sixth People's Hospital, Shanghai, China

**Keywords:** elderly, proximal humeral fracture, regional anesthesia, thoracic paravertebral block, brachial plexus block

## Abstract

**Objective:**

This study was designed to investigate whether it is useful and necessary to add a T2 level thoracic paravertebral block (TPVB) based on brachial-cervical plexus block to avoid incomplete anesthesia in elderly patients undergoing deltopectoral approach proximal humeral fracture (PHF) surgery.

**Materials and Methods:**

This study involved 80 patients scheduled for PHF surgery who were randomized to receive either IC block (combined interscalene brachial plexus with superficial cervical plexus block) or ICTP block (T2 TPVB supplemented with IC block). The primary outcome was the success rate of regional anesthesia. The patient who experienced incomplete block was administered with intravenous remifentanil for rescue, or conversion to general anesthesia (GA) if remifentanil was still ineffective. Secondary outcomes included requirements of rescue anesthesia, sensory block of the surgical region, the incidence of adverse reactions, and block procedure-related complications.

**Results:**

The success rate of regional anesthesia in the ICTP group was higher compared with the IC group (77.5 vs. 52.5%, *p* = 0.019). Intravenous remifentanil was required in 32.5% of patients in the IC group and 17.5% in the ICTP group, respectively. Conversion to GA was performed in 15% of patients in the IC group and 5% in the ICTP group. Sensory block at the medial proximal upper arm was achieved in 85% of patients in the ICTP group, whereas 10% in the IC group (*p* < 0.001). There was no difference between the groups with respect to the incidence of intraoperative adverse reactions. No block-related complications occurred in either group.

**Conclusion:**

Adding a T2 TPVB is helpful to decrease, but not absolutely avoid the occurrence of incomplete regional anesthesia during PHF surgery in elderly patients. However, considering the potential risks, it is not an ideal option while a minor dose of remifentanil can provide a satisfactory rescue effect.

**Clinical Trial Registration:**

ClinicalTrials.gov, identifier: NCT03919422.

## Introduction

Proximal humeral fracture is common and may account for up to 5% of all fractures in the aging population (1). It will affect quality of life, and even increase mortality (2). Treatments for proximal humeral fracture (PHF) include non-operative care and surgery. For the elderly patients with surgical indications, especially those who are afflicted with severe cardiac or pulmonary comorbidity, the choice of anesthesia is a dilemma. Except for general anesthesia (GA), surgery under regional anesthesia (RA) is also an ideal choice. It may preserve spontaneous respiration and minimize the risk of complications associated with GA (3). However, perfect RA presents a unique challenge to anesthesiologists since the relevant innervation is complicated. No single nerve block technique can cover the entire surgical region (4).

The deltopectoral approach for PHF surgery involves the anterior shoulder and the proximal upper arm. The innervation mainly originates from the cervical plexus and the brachial plexus (5, 6). However, the hybrid technique of the superficial cervical plexus block and the interscalene block, which is an effective mode of RA in clavicle and shoulder surgery (7–9), cannot always provide sufficient surgical anesthesia for the patients undergoing PHF surgery. In previous studies, the branches stem from the upper thoracic nerves (T1–T2, even T3), nearly impossible to be covered by a brachial plexus block, were considered to contribute to the innervation of the upper arm as well (10, 11). Zhao (12) also recommended the T2 thoracic paravertebral block (TPVB) as a supplement for RA in PHF surgery since the thoracic paravertebral space (TPVS) houses the ventral rami of nerve roots and allows local anesthetic to spread to T1 and T3. However, limited evidence is available from randomized controlled trials. Therefore, we aimed to investigate whether adding a T2 TPVB is useful and necessary to avoid the occurrence of incomplete regional anesthesia in elderly patients undergoing deltopectoral approach PHF surgery.

## Materials and Methods

This randomized, observer-blinded, controlled trial was approved by the Ethics Committee of Shanghai Sixth People's Hospital (No. 2019-030, March 28, 2019, Chairperson Prof Weiping Jia) and prospectively registered at ClinicalTrials.gov (NCT03919422, April 14, 2019) before the enrolment process started on May 5, 2019.

The patients aged 65 or older, ASA grade I-II, BMI <30 kg/m^2^, scheduled for elective deltopectoral approach PHF surgery between May 2019 and August 2020 were recruited. Exclusion criteria included a request for GA, inability to cooperate, contraindication to RA, multiple traumas, uncontrolled systemic diseases, and allergy to ropivacaine. An independent investigator (HZ) generated the randomized allocation sequence and concealed the allocation results in opaque sealed envelopes. After providing written informed consent, the participants were randomly and equally allocated to receive either combined interscalene brachial plexus with superficial cervical plexus block (IC group) or IC block supplemented with T2 TPVB (ICTP group). On the surgical day, an anesthetic nurse provided one envelope per patient to the anesthesiologist who administered the regional block (XW). After the regional block was finished, this anesthesiologist had no further role in the subsequent procedure. The surgeons, nurses, research assistants, intraoperative anesthesiologists, and outcome assessors were all kept blinded to the allocation.

### Pre-anesthetic Preparation

All the patients underwent preoperative fasting. After standard monitors were applied, intravenous access was established. No sedative or intravenous narcotic was given before RA. Patients were placed in the lateral decubitus position with the operative side upward. An experienced anesthesiologist (XW) performed the nerve block under an aseptic condition using a SonoSite S-Nerve^TM^ ultrasound machine (Bothell, WA, USA) in a dedicated block procedure room. Local lidocaine (1%) for skin numbing was given before each block procedure. The concentration and volume of ropivacaine (Naropin^TM^, AstraZeneca AB, Sweden) for the elderly patients was determined according to the consensus guidelines on RA in China (2014).

### IC Block Procedure

A linear array transducer (6–13 MHz) was positioned on the supraclavicular fossa to locate the supraclavicular brachial plexus. By moving the transducer cranially, the brachial plexus was successively revealed between anterior and middle scalene muscles. A 5-cm, 22-gauge (G) block needle (KDL^TM^, Kindly group, China) was introduced into the plexus sheath with an in-plane approach. Then, 20 ml of 0.375% ropivacaine was injected around the brachial plexus (6). The transducer was then transversely positioned at the mid-point of the posterior edge of the sternocleidomastoid muscle. Furthermore, 10 ml of 0.25% ropivacaine was injected into the fascial plane deep into the muscle (13).

### T2 TPVB Procedure

In the ICTP group, the T_2−3_ intervertebral space was initially identified by both ultrasound image scanning and palpation counting from the C7 spinous process. A curved array transducer (2–5 MHz) was placed with a slightly oblique scan to visualize the T2 transverse process, costotransverse ligament, internal intercostal membrane, and parietal pleura. A 10-cm, 22G needle was inserted into the skin and advanced carefully until the needle tip penetrated the superior costotransverse ligament and positioned in the TPVS. It is a wedge-shaped space beyond the internal intercostal membrane (continuous with superior costotransverse ligament medially) and lateral to the vertebral body on the ultrasound image. Following negative aspiration of air, blood, or cerebrospinal fluid, 10 ml of 0.25% ropivacaine was injected and anterior displacement of the parietal pleura was monitored (14).

### Intraoperative Management

The patient was transferred to the operating room within 10 min after the sensory assessment and placed in a beach-chair position. Each of the patients received preoperative intravenous sedation (1 mg midazolam) and nasal oxygen (3 L/min). Remifentanil (50 μg/ml), propofol (10 mg/ml), and laryngeal mask airway (LMA) were prepared. The regional anesthetic effect was considered to be successful if surgical anesthesia was achieved, otherwise, it is considered to be inadequate if the patient resorts to rescue anesthesia. The rescue procedure would be administered in the following two steps. First, remifentanil would be infused intravenously for 2 min at the rate of 0.25 μg/kg/min. This dosage was defined as a minor dose, which is recommended for elderly patients over 65 according to the package insert. Second, if the patients still complained of pain after remifentanil infusion for 2 min, they would be induced with propofol (1.5–2 mg/kg) for converting to GA with LMA, and maintained with inhaled sevoflurane. All adverse reactions and complications were recorded and managed in accordance with the study protocol. Intraoperative mean arterial pressure higher (or lower) than 30% from the baseline value was defined as hypertension (or hypotension). Hypotension was treated with ephedrine 5–10 mg and/or deoxyepinephrine 50–100 μg IV, while hypertension was treated with urapidil 5–10 mg IV. Atropine 0.5 mg IV was administered to treat bradycardia (heart rate <60 beats/min). The vasoactive medications were used incrementally as required. Hypoxia (SpO_2_ decreased to below 95%) was managed *via* facemask assistant spontaneous ventilation or reducing the rate of remifentanil infusion. The block-related complications, such as local anesthetic systemic toxicity, pneumothorax, epidural and intrathecal injections, hematoma, and so on, were recorded and followed up until resolution.

### Outcome Assessment

The primary outcome was the success rate of regional anesthesia without having to resort to rescue anesthesia. Secondary outcomes included: (1) requirements of rescue anesthesia (including IV remifentanil and GA); (2) sensory block assessment of surgical region; (3) incidence of adverse reactions and block-related complications. The sensory block was assessed bilaterally by the pinprick method using a 22G needle 20 min after the regional block. The testing region around the deltopectoral incision was divided into four areas: (A) supraclavicular region; (B) deltoid region; (C) lateral proximal upper arm; and (D) medial proximal upper arm. The results were described using a 3-point rating scale (0 = no block, normal sensation; 1 = partial block, reduced sensation; 2 = complete block, no sensation at all). An assessor (ZX or YC) blinded to the group allocation collected all the outcome data.

### Sample Size Estimation

Based on the data from our pilot study (*n* = 20, unpublished data), we assumed the success rate to be 60% in the IC group and 90% in the ICTP group, respectively (15). A total sample size of 80 would be required to detect differences with 80% power and a two-tailed alpha error of 5% including the possible dropouts (20%).

### Statistical Analysis

Statistical analyses were performed with SPSS (Version 24, IBM Corporation, Armonk, NY, USA). We used the Kolmogorov-Smirnov test to confirm the normality of data distribution. Continuous data were presented as mean (*SD*) or median (IQR) according to the results of the normal distribution test. The normally distributed numerical data were compared using an independent sample *t*-test, whereas non-parametric data were compared using the Mann-Whitney U-test. Categorical data were presented as numbers (percentages) and compared using the chi-square test or Fisher's exact test. The level of statistical significance was determined at *p* < 0.05.

## Results

**Figure 1:**
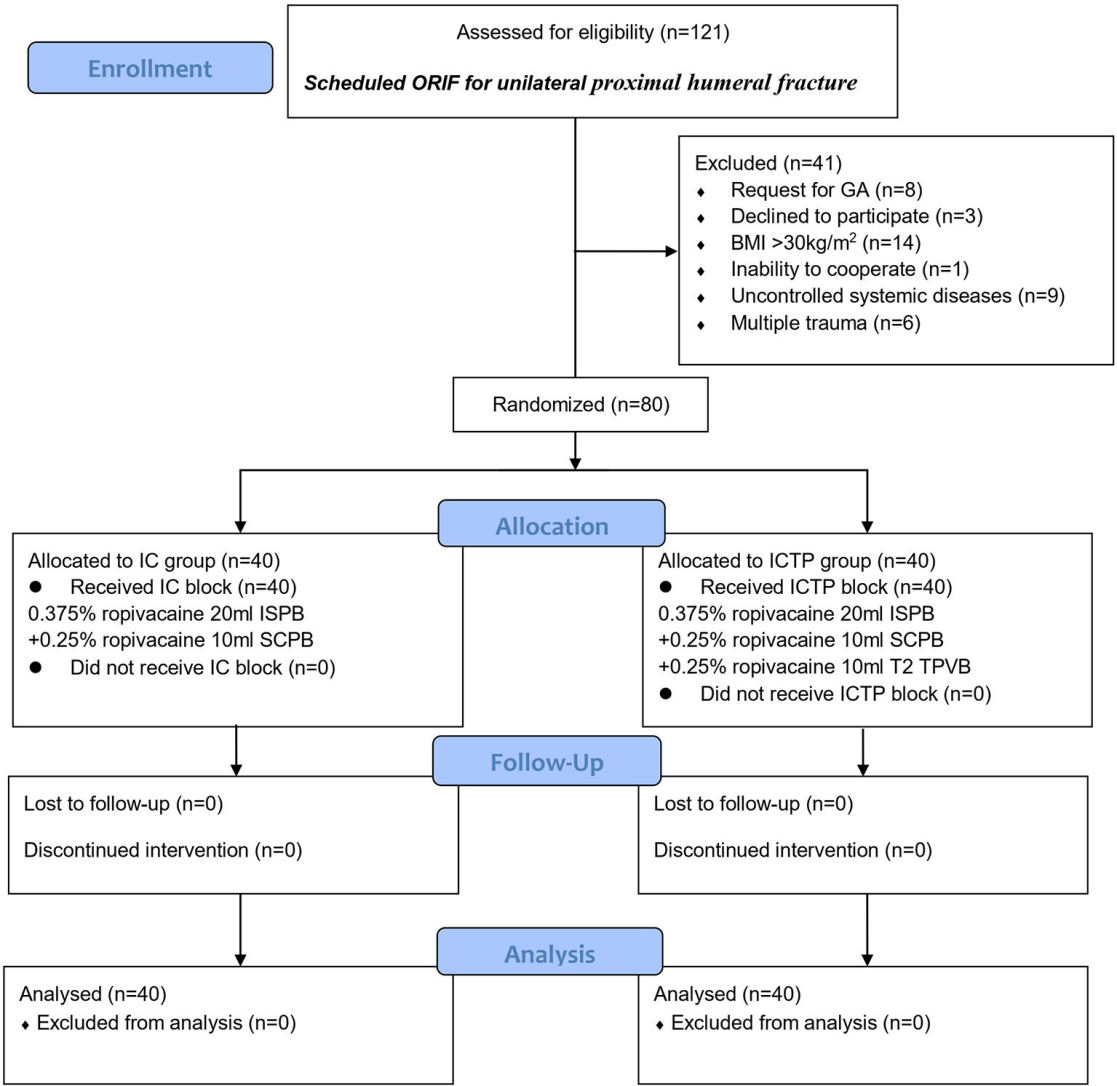
The CONSORT flow diagram. BMI, body mass index; SCPB, superficial cervical plexus block; GA, general anesthesia; IC, combined ISPB with SCPB; ICTP, IC block combined with T2 TPVB; ISPB, interscalene brachial plexus; ORIF, open reduction and internal fixation; TPVB, thoracic paravertebral block.

Among 41 eligible patients, a total of 80 patients were included and divided equally into two groups and all patients received the interventions. The Consolidated Standards Of Reporting Trials (CONSORT) flow diagram is depicted in Figure 1. The baseline demographic data show no statistically significant differences between the two groups (Table 1).

**Table 1:** Baseline demographic characteristics.

	**IC (*n* = 40)**	**ICTP (*n* = 40)**	***p* value**
Age (years)	74.8 ± 6.0	74.5 ± 6.5	0.831
Gender (female/male)	34/6	28/12	0.108
Weight (kg)	60.2 ± 9.1	62.5 ± 11.3	0.316
Height (cm)	157.2 ± 6.8	159.7 ± 7.2	0.114
BMI (kg/m^2^)	24.3 ± 3.2	24.4 ± 3.8	0.927
ASA (I/II)	14/26	17/23	0.491
Duration of surgery (mins)	72.5 (46.3–93.8)	70.0 (56.3–90.0)	0.658

*The values are presented as mean ± SD or median (IQR).*

*ASA, American Society of Anesthesiologists; BMI, body mass index*.

**Figure 2:**
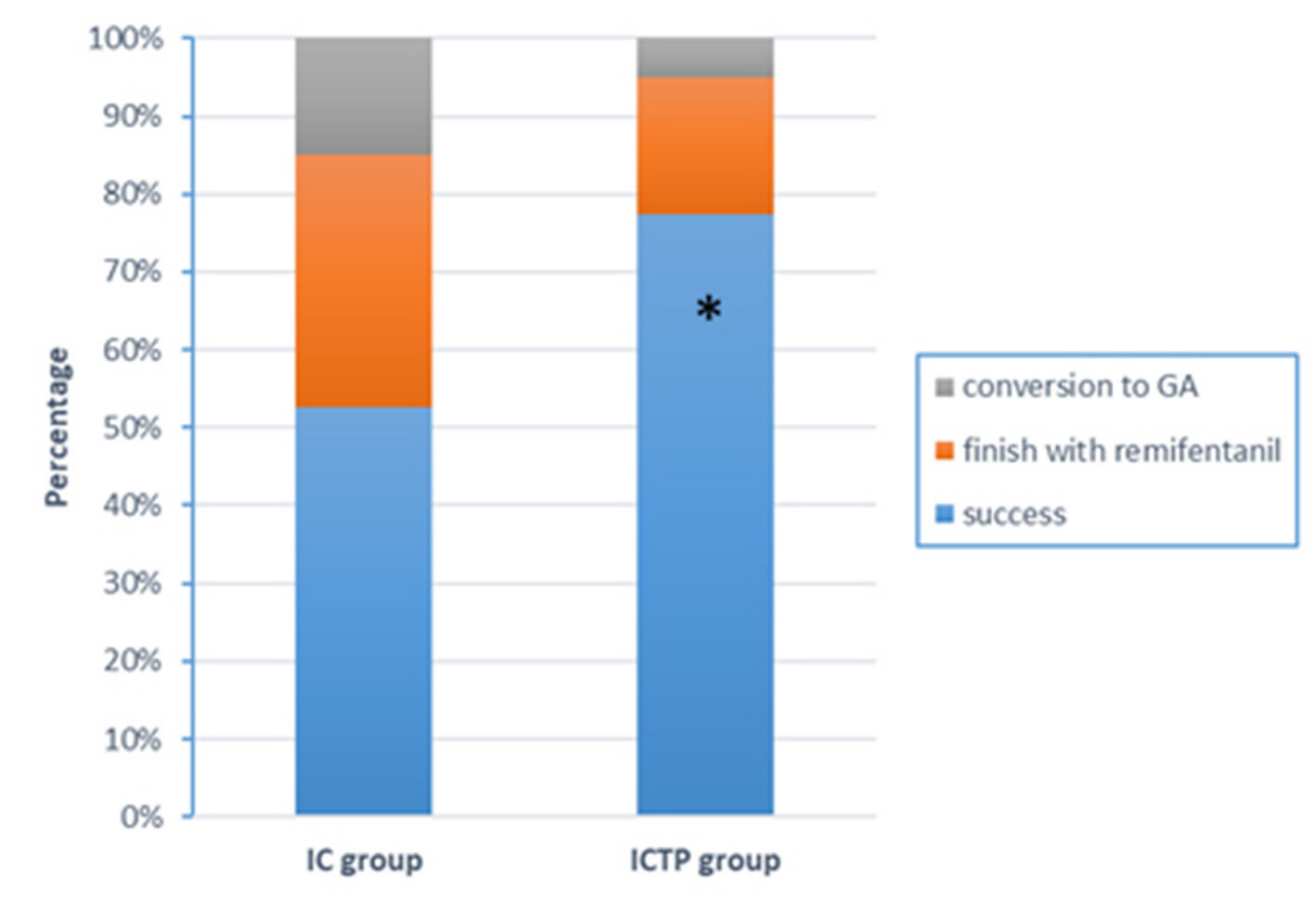
Anesthetic effects outcomes. The success rate of surgical anesthesia in the ICTP group was higher compared with the IC group (77.5 vs. 52.5%, *p* = 0.019). There was no difference in requirement of rescue anesthesia (IV remifentanil or GA) between groups. *p < 0.05 was taken to indicate statistical significance.

The success rate of regional anesthesia in the ICTP group was higher compared with the IC group (77.5 vs. 52.5%, *p* = 0.019, Figure 2). Remifentanil was required and effective in 32.5% of patients in IC group, while 17.5% in ICTP group (*p* > 0.05). Conversion to GA was performed in 15% of patients in the IC group and 5% in the ICTP group, respectively (*p* > 0.05).

**Figure 3:**
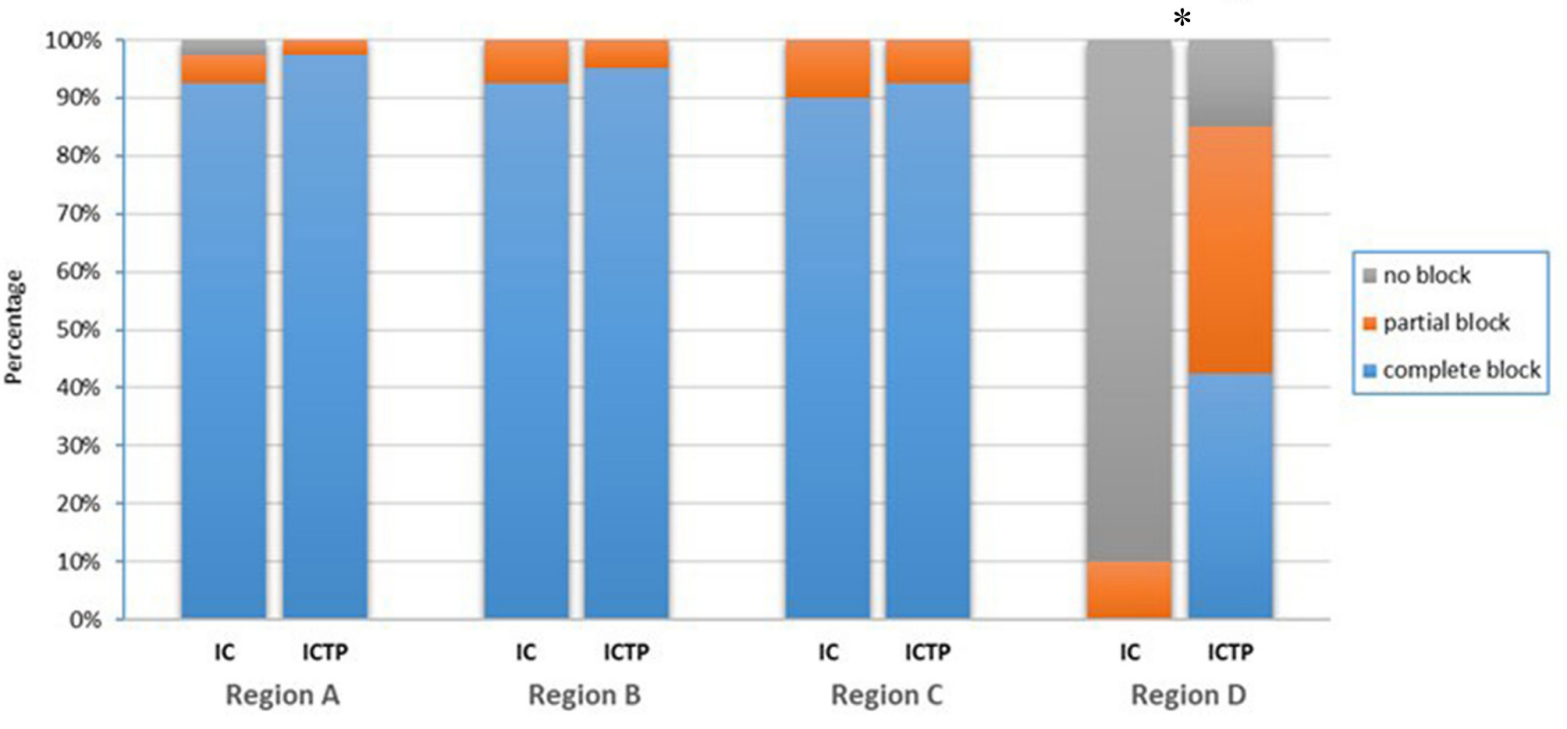
Sensory block outcomes. The result was described as three levels: no block (normal sensation), partial block (reduced sensation), and complete block (no sensation at all). The testing region around the deltopectoral incision was divided into 4 sections: A, supraclavicular region; B, deltoid region; C, lateral proximal upper arm; D, medial proximal upper arm. Sensory block at the medial proximal upper arm (including partial and complete block) was achieved in 85% of patients in the ICTP group, whereas only 10% in the IC group (*p* < 0.001). There was no difference between groups in the other regions. *p < 0.05 was taken to indicate statistical significance.

The sensory block at the medial proximal upper arm (including partial and complete block) was achieved in 85% of patients in the ICTP group, whereas only 10% in the IC group (*p* < 0.001). There was no significant difference in the rate of sensory block at the supraclavicular region, deltoid region, or lateral proximal upper arm between groups (*p* > 0.05). The sensory block assessment is summarized in Figure 3. The two study groups had a similar incidence of intraoperative adverse reactions (Table 2). No block procedure-related complications occurred in either group.

**Table 2:** Incidence of adverse reactions.

**Intraoperative adverse**	**IC (*n* = 40)**	**ICTP (*n* = 40)**	***p* value**
**reactions (%)**			
Hypertension	6 (15)	7 (17.5)	0.762
Hypotension	4 (10)	5 (12.5)	1.000
Bradycardia	5 (12.5)	8 (20)	0.363
Tachycardia	0 (0)	1 (2.5)	0.500
Hypoxia	5 (12.5)	2 (5)	0.429

*Values are presented as absolute numbers (percentage, %)*.

## Discussion

The results of our study indicated that supplementary T2 TPVB was helpful to decrease, but not absolutely avoid the occurrence of incomplete RA. With the assistance of a minor dose of remifentanil, the majority of patients avoided conversion to GA. These findings provide us evidence to discuss the pending question of whether it is necessary to perform an additional upper thoracic nerve level block with the cervical-brachial plexus block-based regional anesthesia during PHF surgery.

The deltopectoral approach covers from the anterior shoulder to the proximal upper arm. The innervation (including dermatome, myotome, and sclerotome) mainly involves supraclavicular nerve (C3–C4), suprascapular nerve (C5–C6), axillary nerve (C5–C6), lateral pectoral nerve (C5–C7), and musculocutaneous nerve (C5–C7) (16–18). These nerves originate from the cervical and brachial plexus, but their contributions vary across individuals. In the present study, the superficial cervical and interscalene brachial plexus blocks provided complete surgical anesthesia for merely 52.5% of the patients in the IC group. It means that this surgical region is not consistently innervated by the above nerves. Relatively, with the supplement of T2 TPVB, 77.5% of the patients in the ICTP group did not require rescue anesthesia. Although this was not an ideal outcome for clinical application even achieved statistic difference, it at least proved that adding a T2 TPVB was beneficial to improve the regional anesthetic effect in part of the population.

The reason for incomplete regional anesthesia possibly is multifactorial. Firstly, the inferior trunk perhaps was not thoroughly blocked due to the fact that the interscalene block mainly targets the superior and middle trunks. The C8 root is difficult to be absolutely blocked by the spread of LA during the interscalene groove or TPVS (19). Therefore, the combination mode of different approaches should be improved for a more comprehensive brachial plexus block. The hybrid technique of interscalene and supraclavicular approach, interscalene and C8 block, or selective trunks block was recently proposed to completely block the three brachial trunks in PHF surgery (11, 20–22). However, none of the modes was supported by neither the large cohort of patients nor the randomized controlled trial. Interestingly, the authors also mentioned that the above techniques were unable to provide a sensory block to the medial aspect of the proximal upper arm due to the missing block of T2–T3. Regrettably, none of them proposed adding an upper thoracic nerve block in PHF surgery. Secondly, T2 TPVB failed to block the sensory of the medial proximal upper arm in 15% of patients, which might be attributable to the unexpected spread of local anesthetic (LA). In a previous study, the researchers administered TPVB at the T3 level and found that the LA mainly spread to T3–T5 rather than T2 (23). The study of Naja and Lönnqvist (24) also proved that sensory spread was usually observed below the level of injection, with very limited cephalad spread. The study conducted by Ruscio et al. (25) concluded that the sagittal approach TPVB had a higher success rate than both transversal in-plane and out-of-plane techniques (93/81/83%). In a cadaver study, a higher injection volume resulted in a larger number of stained thoracic nerves (26). Thus, a modified TPVB technique based on volume or approach might provide a more successful RA.

Under this situation, to weigh the pros and cons, the risks of TPVB should be taken into account. In our study, the incidences of adverse reactions were not significantly different between the two groups, although seemed a little higher compared with the previous studies (27–29). This slight discrepancy might be due to the sensibility of remifentanil in the elderly (30). As the method described by Pangthipampai et al. (31), we also injected the LA into the apex of TPVS at the level of the transverse process, which is far from the intervertebral foramen. This will help to prevent unintentional epidural spread and neuraxial complications. However, zero block-related complication does not mean no potential risk. The necessity of adding a T2 TPVB should be considered seriously since a minor dose of remifentanil infusion was also an effective rescue option. To anxious patients, GA with LMA is more acceptable rather than TPVB or opioids.

We acknowledge some limitations of this study. Firstly, all the block procedures were performed by a single anesthesiologist. This limited the generalizability of our findings, although it reduced performance bias. Secondly, to reduce the incidences of diaphragmatic paralysis and pneumothorax, the combination use of supraclavicular approach and interscalene approach brachial plexus block was not adopted in our study, which might be relevant to the insufficient anesthesia. Lastly, to evaluate the risks of T2 TPVB in elderly patients, we excluded the young population. This might lead to selection bias.

## Conclusion

In conclusion, supplementary T2 TPVB is useful to avoid insufficient regional anesthesia during PHF surgery in some elderly patients. However, considering the potential risks, it is not an ideal option while a minor dose of remifentanil can provide a satisfactory rescue effect. Whether to apply this technique should be decided according to the situation of the patient and the experience of the anesthesiologist.

## Data Availability Statement

The raw data supporting the conclusions of this article will be made available by the authors, without undue reservation.

## Ethics Statement

The studies involving human participants were reviewed and approved by Ethics Committee of Shanghai Sixth People's Hospital. The patients/participants provided their written informed consent to participate in this study.

## Author Contributions

JZ and WJ conceived and led the study design. XW prepared the draft of this manuscript and performed the regional block procedures. HZ administered the randomization and group allocation. QZ recruited the participants. ZX and YC collected the outcome data. JL analyzed the data. All authors read and approved the final version of the manuscript.

## Funding

This trial was supported by the Research Foundations of Shanghai Municipal Health Commission (Nos. 202040257 and 201940510).

## Conflict of Interest

The authors declare that the research was conducted in the absence of any commercial or financial relationships that could be construed as a potential conflict of interest.

## Publisher's Note

All claims expressed in this article are solely those of the authors and do not necessarily represent those of their affiliated organizations, or those of the publisher, the editors and the reviewers. Any product that may be evaluated in this article, or claim that may be made by its manufacturer, is not guaranteed or endorsed by the publisher.

## References

[1] Bell JE Leung BC Spratt KF Koval KJ Weinstein JD Goodman DC. Trends and variation in incidence, surgical treatment, and repeat surgery of proximal humeral fractures in the elderly. J Bone Jt Surg Ser A. (2011) 93:121–31. 10.2106/JBJS.I.01505 21248210 PMC3016042

[2] Bergdahl C Wennergren D Möller M Ekelund J. Mortality after a proximal humeral fracture. Bone Jt J. (2020) 102-B:1484–90. 10.1302/0301-620X.102B11.BJJ-2020-0627.R1 33135440

[3] Girón-Arango L Perlas A. Surgical anesthesia for proximal arm surgery in the awake patient. Reg Anesth Pain Med. (2020) 46:446–451. 10.1136/rapm-2020-101929 33443198

[4] Galluccio F Fajardo Perez M Yamak Altinpulluk E Hou J-D Lin J-A. Evaluation of interfascial plane and pericapsular nerve blocks to the shoulder joint: a preliminary analysis of shoulder anterior capsular block. Pain Ther. (2021) 10:1741–54. 10.1007/s40122-021-00326-0 34669181 PMC8586108

[5] Nadeau M-J Lévesque S Dion N. Ultrasound-guided regional anesthesia for upper limb surgery. Can J Anaesth. (2013) 60:304–20. 10.1007/s12630-012-9874-6 23377861

[6] El-Boghdadly K Chin KJ Chan VWS. Phrenic nerve palsy and regional anesthesia for shoulder surgery: anatomical, physiologic, and clinical considerations. Anesthesiology. (2017) 127:173–91. 10.1097/ALN.0000000000001668 28514241

[7] Arjun BK Vinod CN Puneeth J Narendrababu MC. Ultrasound-guided interscalene block combined with intermediate or superficial cervical plexus block for clavicle surgery: a randomised double blind study. Eur J Anaesthesiol. (2020) 37:979–83. 10.1097/EJA.0000000000001300 32833851

[8] Abdelghany MS Ahmed SA Afandy ME. Superficial cervical plexus block alone or combined with interscalene brachial plexus block in surgery for clavicle fractures: a randomized clinical trial. Minerva Anestesiol. (2021) 87:523–32. 10.23736/S0375-9393.21.14865-5 33591139

[9] Vijayakumar V Ganesamoorthi A Subramaniyan N Kasirajan P. Ultrasound-guided superior and middle trunk brachial plexus block with superficial cervical plexus block for shoulder surgeries in high-risk patients: case series. J Med Ultrasound. (2020) 28:185–7. 10.4103/JMU.JMU_73_19 33282665 PMC7709537

[10] Loukas M El-Zammar D Tubbs RS Apaydin N Louis RG Wartman C. A review of the T2 segment of the brachial plexus. Singapore Med J. (2010) 51:464–7. 20658104

[11] Mistry T Kuppusamy E. Reply to Tognù et al. Regional anesthesia for proximal humerus surgery during COVID-19 pandemic. Reg Anesth Pain Med. (2020) 46:375–6. 10.1136/rapm-2020-101729 32487704

[12] Zhao DQ. Anatomy and Practice of Ultrasound Guided Regional Anesthesia. Beijing: China Population Publishing House (2020).

[13] Senapathi TGA Widnyana IMG Aribawa IGNM Wiryana M Sinardja IK Nada IKW. Ultrasound-guided bilateral superficial cervical plexus block is more effective than landmark technique for reducing pain from thyroidectomy. J Pain Res. (2017) 10:1619–22. 10.2147/JPR.S138222 28761368 PMC5516880

[14] Taketa Y Irisawa Y Fujitani T. Comparison of analgesic efficacy between two approaches of paravertebral block for thoracotomy: a randomised trial. Acta Anaesthesiol Scand. (2018) 62:1274–9. 10.1111/aas.13216 30047132

[15] Nguyen HC Fath E Wirtz S Bey T. Transscalene brachial plexus block: A new posterolateral approach for brachial plexus block. Anesth Analg. (2007) 105:872–5. 10.1213/01.ane.0000271916.26357.8d 17717253

[16] Conroy PH Awad IT. Ultrasound-guided blocks for shoulder surgery. Curr Opin Anaesthesiol. (2011) 24:638–43. 10.1097/ACO.0b013e32834c155f 21934495

[17] Nam YS Panchal K Kim IB Ji JH Park MG Park SR. Anatomical study of the articular branch of the lateral pectoral nerve to the shoulder joint. Knee Surgery Sport Traumatol Arthrosc. (2016) 24:3820–7. 10.1007/s00167-015-3703-8 26194117

[18] Mian A Chaudhry I Huang R Rizk E Tubbs RS Loukas M. Brachial plexus anesthesia: a review of the relevant anatomy, complications, and anatomical variations. Clin Anat. (2014) 27:210–21. 10.1002/ca.22254 23959836

[19] Zisquit J Novella NNN. Interscalene Block - PubMed. Treasure Island, FL: StatPearls (2020). Available online at: https://pubmed.ncbi.nlm.nih.gov/30137775/ (accessed November 5, 2020).

[20] Tognù A Barbara E Pacini I Bosco M. Proximal humeral fracture surgery in the COVID-19 pandemic: advocacy for regional anesthesia. Reg Anesth Pain Med. (2020) 46:375–6. 10.1136/rapm-2020-101626 32409516

[21] Sivakumar RK Areeruk P Karmakar MK. Selective trunk block (SeTB): a simple alternative to hybrid brachial plexus block techniques for proximal humeral fracture surgery during the COVID-19 pandemic. Reg Anesth Pain Med. (2020) 46:376–8. 10.1136/rapm-2020-101733 32522860

[22] Kim E Choi CH Kim JH. Effects of C8 nerve root block during interscalene brachial plexus block on anesthesia of the posterior shoulder in patients undergoing arthroscopic shoulder surgery: study protocol for a prospective randomized parallel-group controlled trial. Trials. (2019) 20:1–9. 10.1186/s13063-019-3624-9 31455407 PMC6712618

[23] Kulhari S Bharti N Bala I Arora S Singh G. Efficacy of pectoral nerve block versus thoracic paravertebral block for postoperative analgesia after radical mastectomy: a randomized controlled trial. Br J Anaesth. (2016) 117:382–6. 10.1093/bja/aew223 27543533

[24] Naja Z Lönnqvist PA. Somatic paravertebral nerve blockade: incidence of failed block and complications. Anaesthesia. (2001) 56:1184–8. 10.1046/j.1365-2044.2001.02084-2.x 11736777

[25] Ruscio L Renard R Lebacle C Zetlaoui P Benhamou D Bessede T. Thoracic paravertebral block: comparison of different approaches and techniques. A study on 27 human cadavers. Anaesth Crit Care Pain Med. (2020) 39:53–8. 10.1016/j.accpm.2019.04.003 30978401

[26] Seidel R Wree A Schulze M. Thoracic-paravertebral blocks: comparative anatomical study with different injection techniques and volumes. Reg Anesth Pain Med. (2020) 45:102–6. 10.1136/rapm-2019-100896 31678963

[27] Pace MM Sharma B Anderson-Dam J Fleischmann K Warren L Stefanovich P. Ultrasound-guided thoracic paravertebral blockade: a retrospective study of the incidence of complications. Anesth Analg. (2016) 122:1186–91. 10.1213/ANE.0000000000001117 26756911

[28] Yeung JH Gates S Naidu BV Wilson MJ Gao Smith F. Paravertebral block versus thoracic epidural for patients undergoing thoracotomy. Cochrane Database Syst Rev. (2016) 2016:CD009121. 10.1002/14651858.CD009121.pub2 26897642 PMC7151756

[29] Schnabel A Reichl SU Kranke P Pogatzki-Zahn EM Zahn PK. Efficacy and safety of paravertebral blocks in breast surgery: a meta-analysis of randomized controlled trials. Br J Anaesth. (2010) 105:842–52. 10.1093/bja/aeq265 20947592

[30] Chaumeron A Castanie J Fortier LP Basset P Bastide S Alonso S. Efficacy and safety of remifentanil in a rapid sequence induction in elderly patients: a three-arm parallel, double blind, randomised controlled trial. Anaesth Crit Care Pain Med. (2020) 39:215–20. 10.1016/j.accpm.2019.09.010 31614244

[31] Pangthipampai P Karmakar MK Songthamwat B Pakpirom J Samy W. Ultrasound-guided multilevel thoracic paravertebral block and its efficacy for surgical anesthesia during primary breast cancer surgery. J Pain Res. (2020) 13:1713–23. 10.2147/JPR.S246406 32765047 PMC7367918

